# Adherence to Smoking Cessation Policy in Inpatient Psychiatric Services at Hamad Hospital: A Retrospective Clinical Audit

**DOI:** 10.1192/bjo.2026.11562

**Published:** 2026-06-30

**Authors:** Sazgar Hamad, Ahmad Alater, Yousaf Iqbal, Pratyaksha Sinha, Menatalla Ali

**Affiliations:** Hamad Medical Corporation, Doha, Qatar

## Abstract

**Aims:**

Tobacco use remains highly prevalent among individuals with severe mental illness and is a major contributor to excess physical morbidity and premature mortality. Institutional smoking cessation policies aim to standardise nicotine dependence assessment, nicotine replacement therapy (NRT) prescribing and monitoring, patient education, and dischargeplanning in psychiatric inpatient settings. However, local data evaluating adherence to these standards in routine clinical practice are limited. This audit aimed to assess adherence to the institutional Smoking Cessation Policy across inpatient psychiatric services at Hamad Hospital, with a focus on documentation of nicotine dependence, NRT prescribing and monitoring, patient education, referral practices, and discharge continuity. It was hypothesised that documentation quality and continuity-of-care components would demonstrate lower compliance than initial screening processes.

**Methods:**

A retrospective clinical audit was conducted between January and August 2024 across inpatient psychiatric units at Hamad Hospital. A total of 144 inpatient medical records were reviewed using the Cerner electronic health record system. Audit standards were derived from the institutional Smoking Cessation Policy and included documentation of smoking status, nicotine dependence screening and severity assessment, initiation and monitoring of NRT, documentation of side effects, delivery of patient and family education, provision of motivational interventions, referral to the Tobacco Control Center, and smoking cessation planning within discharge summaries. Data were extracted using a standardised audit tool and analysed descriptively.

**Results:**

Approximately 40% of admitted patients were identified as current smokers. Nursing compliance with initial nicotine dependence screening was high, exceeding 97%. In contrast, documentation of nicotine dependence severity was recorded in only 30.2% of cases. Appropriate NRT strength was documented in 12.3% of smokers, while monitoring for NRT-related side effects was not documented in any record. Only 20% of identified smokers received NRT, while 29% declined treatment. Referral to the Tobacco Control Center was documented in 18% of cases, with a further 13% documenting patient refusal. Motivationalinterventions were documented in 1.8% of cases. No records contained documentation of patient or family education or provision of educational materials. Discharge summaries showed limited continuity planning, with NRT prescriptions documented in only 3.3% of cases.

**Conclusion:**

This audit demonstrates significant gaps between institutional smoking cessation policy standards and routine clinical practice within psychiatric inpatient services, particularly in documentation quality, patient education, and discharge continuity. While initial screening processes were consistently completed, downstream interventions and continuity-of-care measures were poorly documented. Targeted staff training, improved access to educational resources, and structured monitoring and documentation systems are required to strengthen implementation of smoking cessation policy and improve tobacco-related health outcomes for psychiatric inpatients.

